# Flavokawain C inhibits glucose metabolism and tumor angiogenesis in nasopharyngeal carcinoma by targeting the HSP90B1/STAT3/HK2 signaling axis

**DOI:** 10.1186/s12935-024-03314-4

**Published:** 2024-05-06

**Authors:** YuQiang Hu, ChenJie Yu, LiangJun Cheng, Chang Zhong, Jun An, MingZhen Zou, Bing Liu, Xia Gao

**Affiliations:** 1 Department of Otolaryngology Head and Neck Surgery, Drum Tower Clinical Medical College, Nanjing Medical University, No.321, Zhongshan Road, Nanjing, 210008 Jiangsu China; 2 Department of Otolaryngology Head and Neck Surgery, XuZhou Central Hospital, (Xuzhou Clinical School of Nanjing Medical University), No.199, Jiefang South Roa, Xuzhou, 221009 Jiangsu China; 3 Department of Otolaryngology Head and Neck Surgery, Xuzhou Clinical School of Xuzhou Medical University, Xuzhou, 221004 Jiangsu China

**Keywords:** Nasopharyngeal carcinoma, Flavokawain C, HSP90B1, EGFR, PI3K/Akt/mTOR signaling axis

## Abstract

**Objective:**

Over the past decade, heat shock protein 90 (HSP90) inhibitors have emerged as promising anticancer drugs in solid and hematological malignancies. Flavokawain C (FKC) is a naturally occurring chalcone that has been found to exert considerable anti-tumor efficacy by targeting multiple molecular pathways. However, the efficacy of FKC has not been studied in nasopharyngeal carcinoma (NPC). Metabolic abnormalities and uncontrolled angiogenesis are two important features of malignant tumors, and the occurrence of these two events may involve the regulation of HSP90B1. Therefore, this study aimed to explore the effects of FKC on NPC proliferation, glycolysis, and angiogenesis by regulating HSP90B1 and the underlying molecular regulatory mechanisms.

**Methods:**

HSP90B1 expression was analyzed in NPC tissues and its relationship with patient’s prognosis was further identified. Afterward, the effects of HSP90B1 on proliferation, apoptosis, glycolysis, and angiogenesis in NPC were studied by loss-of-function assays. Next, the interaction of FKC, HSP90B1, and epidermal growth factor receptor (EGFR) was evaluated. Then, in vitro experiments were designed to analyze the effect of FKC treatment on NPC cells. Finally, in vivo experiments were allowed to investigate whether FKC treatment regulates proliferation, glycolysis, and angiogenesis of NPC cells by HSP90B1/EGFR pathway.

**Results:**

HSP90B1 was highly expressed in NPC tissues and was identified as a poor prognostic factor in NPC. At the same time, knockdown of HSP90B1 can inhibit the proliferation of NPC cells, trigger apoptosis, and reduce glycolysis and angiogenesis. Mechanistically, FKC affects downstream EGFR phosphorylation by regulating HSP90B1, thereby regulating the phosphatidylinositol 3-kinase (PI3K)/protein kinase B (Akt)/mammalian target of rapamycin (mTOR) pathway. FKC treatment inhibited the proliferation, glycolysis, and angiogenesis of NPC cells, which was reversed by introducing overexpression of HSP90B1. In addition, FKC can affect NPC tumor growth and metastasis in vivo by regulating the HSP90B1/EGFR pathway.

**Conclusion:**

Collectively, FKC inhibits glucose metabolism and tumor angiogenesis in NPC by targeting the HSP90B1/EGFR/PI3K/Akt/mTOR signaling axis.

**Supplementary Information:**

The online version contains supplementary material available at 10.1186/s12935-024-03314-4.

## Introduction

Nasopharyngeal carcinoma (NPC) is a typical head and neck malignant tumor that originates from the epithelium of human nasopharyngeal mucosa with a relatively high degree of malignancy [1]. The mortality rate of NPC in China accounts for 2.81% of the national malignant tumor mortality rate, and the morbidity rate accounts for more than 80% of the world's [2]. NPC etiology is related to multiple factors, including genetics, geography, diet, and viral infection [3]. Due to insidious symptoms, tumor aggressiveness, and unawareness, most patients are diagnosed with advanced NPC [4], for whom radiotherapy, chemotherapy, or molecular targeted therapy are usually administrated [5]. However, due to the highly malignant, aggressive, and metastatic nature of NPC, the therapeutic effect is not very satisfactory [6]. Therefore, finding effective molecular targets for clinical treatment is of great significance for prolonging patient survival.

Aerobic glycolysis and pathological angiogenesis are the most important hallmarks of cancer. Cancer cells rely heavily on glycolysis for energy metabolism even under normal oxygen concentrations [7] and convert most incoming glucose to lactate, which allows cancer cells to have a growth advantage [8, 9]. To adapt to hypoxia caused by the rapid proliferation of cancer cells, cells upregulate glycolysis, increase acid production, and reduce local extracellular pH [10]. Cancer invasion and angiogenesis can be accelerated after microenvironment acidification by disrupting adjacent normal cells and degrading the extracellular matrix [11]. Moreover, tumor pathological angiogenesis promotes NPC metastasis [12].

Chalcone has been shown to exhibit anticancer activity by targeting a variety of molecular pathways, such as apoptosis, cell cycle, p53 pathway, NF-kappa B, tubulin polymerization, ubiquitin–proteasome pathway, and β-catenin/Wnt [13]. In addition, Chalcone has low toxicity to normal cells [14]. Because of these properties, Chalcone has emerged as a potential candidate for an anticancer or preventative cancer. Flavokawain C (FKC) is a naturally occurring chalcone that belongs to the flavonoid family and can be isolated from the Kava (*Piper methysticum Forst)* root [15]. Recently, FKC has shown considerable efficacy in colon cancer by targeting multiple molecular pathways [16, 17].

Heat shock protein 90 (HSP90), a conserved molecular chaperone, promotes the maturation of oncoproteins in cancer cells, including protein kinases, ribonucleoproteins, steroid hormone receptors, and transcription factors [18]. HSP90 family includes hsp90aa, hsp90ab, hsp90b1, and TRAP [19]. HSP90B1 is located in the endoplasmic reticulum, and its gene exists on chromosome 12q23.3. When endoplasmic reticulum stress occurs, HSP90B1 expression is increased, promoting protein refolding [20]. It is worth noting that HSP90B1, as a tumor-promoting factor, is highly expressed in non-small cell lung cancer [20], bladder cancer [21], and breast cancer [22], which is of great significance for patients’ prognosis. Our previous studies have shown that FKC is a natural inhibitor of HSP90B1, which can effectively reduce HSP90B1 expression in cancer cells. However, the potential downstream pathways and mechanisms by which FKC regulates HSP90B1 in NPC remain unclear.

This study explored the potential molecular mechanism by which FKC regulates HSP90B1 and influences NPC proliferation, glycolysis, and angiogenesis. It was determined that HSP90B1 binds to epidermal growth factor receptor (EGFR) and promotes NPC development by activating the phosphatidylinositol 3-kinase (PI3K)/protein kinase B (Akt)/mammalian target of rapamycin (mTOR) pathway. This provides a new strategy for the development of NPC-targeted drugs and the exploration of therapeutic targets.

## Materials and methods

### NPC clinical sample collection

From 2014 to 2017, we procured 42 pairs of freshly frozen tumor tissues and adjacent normal nasopharyngeal epithelial tissues (situated at a distance greater than 3 cm from the tumor) from patients diagnosed with NPC undergoing treatment at our facility. Furthermore, 5 pairs of formalin-fixed, paraffin-embedded biopsy tissues from NPC and nearby tissues were collected. Prior to biopsy, all patients were histopathologically confirmed to have primary NPC and had not received any oncological treatments. Two NPC experts conducted histological verification of the collected tissues. The study adhered to the principles of the Declaration of Helsinki and received approval from the Ethics Committee of XuZhou Central Hospital. Informed consent was obtained from all subjects, and they were monitored for a follow-up period of five years.

### Immunohistochemistry

The formalin-fixed, paraffin-embedded tissue sections underwent standard deparaffinization and rehydration, followed by antigen retrieval. Blocking of intrinsic peroxidase activity was achieved using 3% hydrogen peroxide at 25 degrees Celsius for 25 min. The sections were incubated with primary antibodies: HSP90B1 (ab3674, Abcam) and EGFR (ab52894, Abcam) at 4 degrees Celsius overnight, and subsequently with secondary antibodies for 90 min at ambient temperature. Imaging of representative areas was done using Aperio ImageScope software version 11.2.0.780 (Leica Biosystems, Wetzlar, Germany).

Quantitative Real-Time Polymerase Chain Reaction (RT-qPCR).

Total ribonucleic acid (RNA) was extracted from the tissues and cells using TRIzol reagent (15,596,018, Thermo Fisher Scientific), then reverse transcribed to complementary DNA (cDNA) using PrimeScript RT master mix (RR058A, Takara, Japan). The RT-qPCR was performed using SYBR Green qPCR Super Mix-UDG (Thermo Fisher Scientific). Glyceraldehyde 3-phosphate dehydrogenase (GAPDH) was employed as the internal reference gene. Gene expression quantification was based on the 2−^ΔΔCT^ method. Primer sequences used in the RT-qPCR are detailed in Table 1.

**Table 1:** RT-qPCR primer sequences

	Primers
HSP90B1	Forward: 5'- GGATGGTCTGGCAACATGGA-3'
	Reverse: 5'- CCGAAGCGTTGCTGTTTCAA-3'
EGFR	Forward: 5'- CGGGCTCTGGAGGAAAAGAA -3'
	Reverse: 5'- CAGCTCCTTCAGTCCGGTTT -3'
GAPDH	Forward: 5'-CACCCACTCCTCCACCTTTG-3'
	Reverse: 5'-CCACCACCCTGTTGCTGTAG-3'

Note: *HSP90B1* heat shock protein 90B1, *EGFR* epidermal growth factor receptor, *GAPDH* glyceraldehyde 3-phosphate dehydrogenase

### Cell culture

Five NPC cell lines, namely HNE1, HNE2, CNE1, CNE2, and HONE1, were cultured in Roswell Park Memorial Institute-1640 medium (Invitrogen, Carlsbad, USA) supplemented with 10% fetal bovine serum (FBS, Gibco, Grand Island, USA). The immortalized human nasopharyngeal epithelial cell line, NP69, was maintained in keratinocyte/serum-free medium (Invitrogen) supplemented with bovine pituitary extract (BD Bioscience, CA, USA). All cell lines, sourced from the Cell Bank of the Chinese Academy of Sciences (Shanghai, China), were incubated in a humidified atmosphere with 5% CO_2_ at 37 °C. The cell lines were authenticated using Short Tandem Repeat profiling by GENEWIZ Biotechnology Co., Ltd. (Suzhou, China) and routinely screened for mycoplasma contamination (Yeasen, # 40601ES20). To assess the impact of the compound FKC on NPC cell biological behavior, the cells were treated with 4 μM FKC for 48 h.

### Cell transfection

The cDNA of HSP90B1 was amplified by PCR and subcloned into the pcDNA3.1 vector (Invitrogen), with the final construct confirmed by sequencing for overexpressing HSP90B1. The empty pcDNA3.1 vector was used as a control. Small interfering RNA (siRNA) targeting HSP90B1 (si-HSP90B1: 5′-atggattaaatgcatcacaaata-3′) and negative control (si-NC) were designed and provided by RiboBio (Guangzhou, China) for knocking down HSP90B1 and EGFR. When cell confluence reached 70%–80%, transfection with the aforementioned plasmids or oligonucleotides was performed using Lipofectamine 3000 (L3000015, Thermo Fisher Scientific) following the manufacturer's protocol. After 48 h, transfection efficiency was assessed by RT-qPCR and Western blot. To inhibit the EGFR signaling pathway, HNE1 and CNE2 cells were treated with cetuximab (Erbitux^®^, Merck KGaA, Darmstadt, Germany, 20 ng/ml).

### Cell counting Kit-8 (CCK-8) assay

To evaluate the cell viability of NP69, cells (4500 cells/well) were seeded into a 96-well plate. Cells were treated with various concentrations of FKC (0.5, 1, 2, 4 μM) under 5% CO_2_ at 37 °C for 48 h. Each well was supplemented with 10 μL CCK-8 solution (Sangon Biotech, Shanghai, China), and incubated for an additional 4 h. The absorbance at 450 nm was measured using an iMark microplate reader (Bio-Rad, Hercules, CA, USA). Cell viability (100%) = (OD of the experimental group—OD of the blank group)/(OD of the control group—OD of the blank group) × 100%.

For assessing cell proliferation, HNE1 and CNE2 cells were treated with different concentrations of FKC (0.5, 1, 2, 4 μM) for 48 h, then harvested and seeded into a 96-well plate (2000 cells/well). Cells were incubated in 96-well plates with 100 µl of complete medium with 5% CO_2_ at 37 °C. At designated time points (24, 48, and 72 h), 10 μL of CCK-8 solution (Sangon Biotech, Shanghai, China) was added to each well, followed by a 2 h incubation. Absorbance at 450 nm was measured using an iMark microplate reader (Bio-Rad) [23].

### Colony formation assay

HNE1 and CNE2 cells were seeded in 6-well plates at a density of 500 cells per well and cultured for 2 weeks. After washing with Phosphate-Buffered Saline, the cells were fixed with 4% paraformaldehyde and stained with 0.5% crystal violet (V5265, Sigma-Aldrich). The number of colonies (diameter ≥ 100 μm) was manually counted to reflect cell proliferative capacity.

### 5-Ethynyl-2'-deoxyuridine (EdU) Proliferation Assay.

Cell proliferation was determined using an EdU assay kit (C10310, RiboBio) according to the manufacturer's instructions. Briefly, treated cells were seeded into 6-well plates at a density of 5 × 10^3^ cells/well and incubated with 100 μl of 50 μM EdU at 37 °C, followed by fixation with 4% paraformaldehyde. After nuclear staining with 4', 6-diamidino-2-phenylindole (DAPI), EdU-positive cells were observed and analyzed under a fluorescence microscope (Olympus, Japan) to reflect changes in cell proliferation. The EdU positive rate was calculated as EdU positive/DAPI × 100%.

### Flow cytometry

For apoptosis assessment, HNE1 and CNE2, NPC cell lines, were harvested and stained using an Annexin V-Fluorescein Isothiocyanate (FITC) Apoptosis Detection Kit (Vazyme) [24, 25]. Suspended and trypsinized cells were washed with ice-cold Dulbecco’s Modified Eagle Medium (DMEM) with Hank’s Balanced Salt Solution (D-Hanks solution). After centrifugation, cells were resuspended in binding buffer, mixed with 5 µL of Annexin V-FITC and 5 µL of propidium iodide, and incubated in the dark at room temperature for 15 min. Apoptotic rates were analyzed using a FACSCalibur flow cytometer (BD Biosciences, San Jose, CA, USA).

### Metabolic assay

The Extracellular Acidification Rate (ECAR) was determined using a Seahorse Biosciences XF96 Analyzer (North Billerica, MA, USA) [26]. HNE1 and CNE2 cells were seeded in XF96 well assay plates and incubated overnight. ECAR was measured under basal conditions and in response to 10 mM glucose, 5 μM oligomycin, and 100 mM 2-deoxyglucose (all from Sigma-Aldrich). The protocol included 3 min of mixing, 3 min of waiting, and 3 min of measurement, with ECAR values reported as milli-pH per minute (mpH/min).

### Glucose consumption and lactate production assay

Glucose and lactate levels in the culture medium of HNE1 and CNE2 cells were quantified using the Glucose Assay Kit and Lactate Assay Kit (Abcam), respectively. Following the manufacturer's instructions, culture medium was incubated with reaction mixtures at room temperature for 30 min, and absorbance at 450 nm was measured using a Bio-Tek microplate reader.

### Human umbilical vein endothelial cell (HUVEC) tube formation assay

Conditioned media from HNE1 and CNE2 cells were collected. Precooled Matrigel (BD Biosciences) was added to precooled 96-well plates (50 µL per well) and allowed to solidify at 37 °C for 30 min. HUVECs were seeded and incubated with conditioned media at 37 °C for 6 h. Tube formation was observed and captured using an inverted microscope (Olympus).

### HUVEC migration assay

HUVECs were harvested and suspended in DMEM without FBS. A 400 µL cell suspension containing 10,000 cells was seeded into the upper chamber of a Millicell insert (Millipore, USA). NPC cell-conditioned medium (600 µL) was added to the lower chamber. After 36 h, cells in the Transwell chambers were fixed with 4% formalin (Sevicebio, Wuhan, China) and stained with 0.1% crystal violet for 30 min. Migrated cells were counted post washing with PBS and imaged using a microscope (Olympus) [27].

### Co-immunoprecipitation (Co-IP) experiment

For Co-IP assays, approximately 10^7^ HNE1 and CNE2 cells were lysed in IP buffer containing 50 mM Tris–HCl pH 7.4, 150 mM Sodium Chloride (NaCl), 2 mM Ethylenediaminetetraacetic Acid, and 1% Nonidet P-40, supplemented with 1 × protease inhibitor mixture and 1 mM Phenylmethylsulfonyl fluoride. Primary antibodies were added to the supernatants of the lysates and incubated for 2 h, followed by the addition of Protein A/G magnetic beads to each sample. The beads were then resuspended in 2 × Loading Buffer and boiled at 95 °C for 10 min. After washing, protein complexes were resolved in Sodium Dodecyl Sulfate (SDS) loading buffer and analyzed by Western blot using antibodies against HSP90B1 (ab3674, Abcam), EGFR (ab52894, Abcam), and IgG (ab37355, Abcam).

### Western blot

Cells and tissues were lysed in radioimmunoprecipitation assay buffer (Beyotime, Jiangsu, China) for protein extraction, and protein concentrations were determined using the bicinchoninic acid Protein Assay. Proteins were separated via SDS–Polyacrylamide Gel Electrophoresis (Beyotime) and transferred to polyvinylidene fluoride membranes (Millipore, Billerica, MA, USA). Membranes were blocked with 5% skim milk at room temperature for 1 h and incubated overnight at 4 °C with primary antibodies against HSP90B1 (ab3674, Abcam), phosphorylated EGFR (p-EGFR, 3777, Cell Signaling Technology), EGFR (ab52894, Abcam), Glucose Transporter 1 (GLUT1, ab652, Abcam), Hexokinase 2 (HK2, 22,029-1-AP, Proteintech), Angiopoietin 1 (Ang-1, ab8451, Abcam), Vascular Endothelial Growth Factor (VEGF, ab46154, Abcam), PI3K (4249, Cell Signaling Technology), phosphorylated PI3K (p-PI3K, 4228, Cell Signaling Technology), Akt (4691, Cell Signaling Technology), phosphorylated Akt (p-Akt, 9271, Cell Signaling Technology), phosphorylated mTOR (p-mTOR, 5536, Cell Signaling Technology), mTOR (2972, Cell Signaling Technology), and Glyceraldehyde 3-Phosphate Dehydrogenase (2118, Cell Signaling Technology). Horseradish Peroxidase-conjugated secondary antibodies were used for further incubation, and immunoblots were visualized using Clarity Max Western ECL Substrate (32,134, Pierce Biotechnology, Rockford, IL, USA).

### Tumor xenograft model in mice

This animal study was approved by the Animal Ethics Committee of XuZhou Central Hospital and conducted in strict accordance with the committee’s guidelines. Sixty male BALB/C nude mice, aged 5–6 weeks, were procured from the Charles River Laboratories (Hunan SJA Laboratory Animal Co., Ltd).

Mice were randomly divided into six groups: PBS group, FKC group, FKC + si-NC group, FKC + si-HSP90B1 group, FKC + pcDNA 3.1 group, and FKC + pcDNA 3.1-HSP90B1 group. The PBS and FKC groups were subcutaneously inoculated with HNE1 cells treated with PBS (2 × 10^6^ cells/mouse), while the other groups received HNE1 cells with either stable knockdown or overexpression of HSP90B1 (2 × 10^6^ cells/mouse). To determine the inhibitory effect of FKC on NPC tumor growth, treatment with FKC (3 mg/kg) was initiated via intraperitoneal injection when tumor volumes reached 70–120 mm^3^ [28]. The PBS group received an equivalent volume of vehicle solution (0.9% saline containing 4% Dimethyl sulfoxide and 5% Tween 80) as a control. Tumor volumes were measured weekly using calipers, with the volume calculated as length × width^2^/2. After 4 weeks, mice were euthanized, and tumor tissues were collected for gene analysis.

To assess the impact of FKC on tumor liver metastasis, HNE1 cells infected with a lentivirus expressing luciferase were used. Post-infection, cells were selected for efficient viral integration and stable luciferase expression using flow cytometry. Mice were intravenously injected with these cells (2 × 10^6^ cells/mouse). Over the subsequent 8 weeks, specific FKC treatment (3 mg/kg) was administered. At the end of the experiment, intraperitoneal injection of luciferin solution (150 mg/kg) was performed for quantitative assessment of tumor metastasis using bioluminescence imaging [29].

### Data analysis

Statistical analysis was conducted using SPSS software version 19.0, GraphPad Prism version 5, and ImageJ software. A two-tailed unpaired Student's t-test was used for comparisons between two groups. One-way Analysis of Variance was applied for multiple group comparisons. Pearson correlation analysis was performed to analyze the correlation between two genes. Survival curves were estimated using the Kaplan–Meier method and compared using the log-rank test. Data are presented as mean ± standard deviation (SD). A P-value of less than 0.05 was considered statistically significant.

## Results

### Elevated expression of HSP90B1 in NPC

**Fig. 1:**
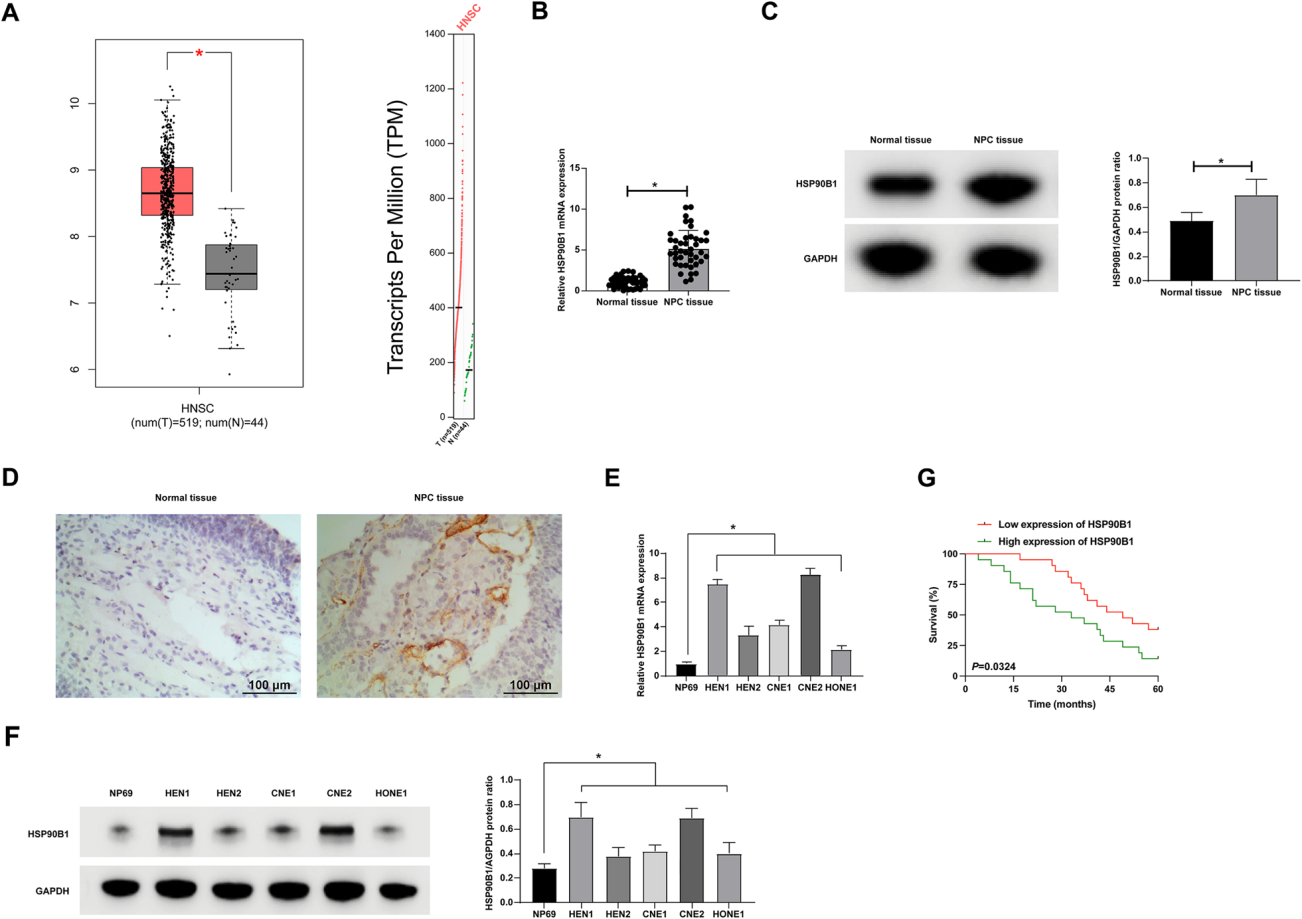
HSP90B1 is overexpressed in NPC. **A**: Bioinformatics website http://gepia.cancer-pku.cn/index.html analyzed the expression parttern of HSP90B1 in HNSC; **B**: RT-qPCR detection of HSP90B1 in NPC tissues and normal nasopharyngeal epithelial samples; **C**: Western blot detection of HSP90B1 expression pattern in NPC tissues and normal nasopharyngeal epithelial samples; **D**: IHC staining to assess the expression pattern of HSP90B1 in NPC tissues and normal nasopharyngeal epithelial samples; **E**: RT-qPCR to assess the expression pattern of HSP90B1 in the NPC cell lines (HNE1, HNE2, CNE1, CNE2, HONE1) and human immortalized nasopharyngeal epithelial cell line (NP69); **F**: Western blot assessment of HSP90B1 expression in NPC cell lines (HNE1, HNE2, CNE1, CNE2, HONE1) and NP69; **G**: Prognostic chart of 5 year survival of 42 NPC patients. Data are expressed as mean ± SD (N = 3). p < 0.05 was considered statistically significant

HSP90B1, a recognized tumor facilitator in several cancers such as NSCLC [20], bladder cancer [21], and breast cancer [22], presents an intriguingly high expression in NPC, the implications of which are yet to be fully understood. Our initial bioinformatic analysis (http://gepia.cancer-pku.cn/index.html) identified a notable overexpression of HSP90B1 in HNSC. Further scrutiny involving 42 NPC patient samples revealed, via RT-qPCR, Western blot, and IHC staining, that HSP90B1 was consistently upregulated in NPC tissues as opposed to normal nasopharyngeal epithelial counterparts (Fig. 1B–D). This pattern was echoed in five distinct NPC cell lines, all exhibiting heightened HSP90B1 expression compared to regular nasopharyngeal epithelial cell lines (Fig. 1E, F). Subdividing NPC patients based on HSP90B1 expression median, we observed a correlation between high HSP90B1 levels and increased incidence of lymph node and distant metastases (Table 2). Kaplan–Meier analysis further highlighted a reduced 5 year survival rate in patients with elevated HSP90B1 expression (Fig. 1G), suggesting its potential role as an oncogenic factor in NPC.

**Table 2:** Relationship between HSP90B1 and clinicopathological features of NPC patients

Characteristics	Cases	The expression of HSP90B1		P
	*n* = 42	Low (*n* = 21)	High (*n* = 21)	
Gender				0.4537
Male	33	15	18	
Female	9	6	3	
Age (year)				
≤ 60	17	7	10	0.5303
> 60	25	14	11	
Tumor size				0.4841
< 3 cm	31	17	14	
≥ 3 cm	11	4	7	
TNM staging				
I/II	28	13	15	0.7442
II/IV	14	8	6	
Lymph node metastasis				0.0247
Positive	16	4	12	
Negative	26	17	9	
Distant metastasis				
Positive	12	2	10	0.0148
Negative	30	19	11	

### Elucidating the role of HSP90B1 in NPC: a triad of proliferation, glycolysis, and angiogenesis

**Fig. 2:**
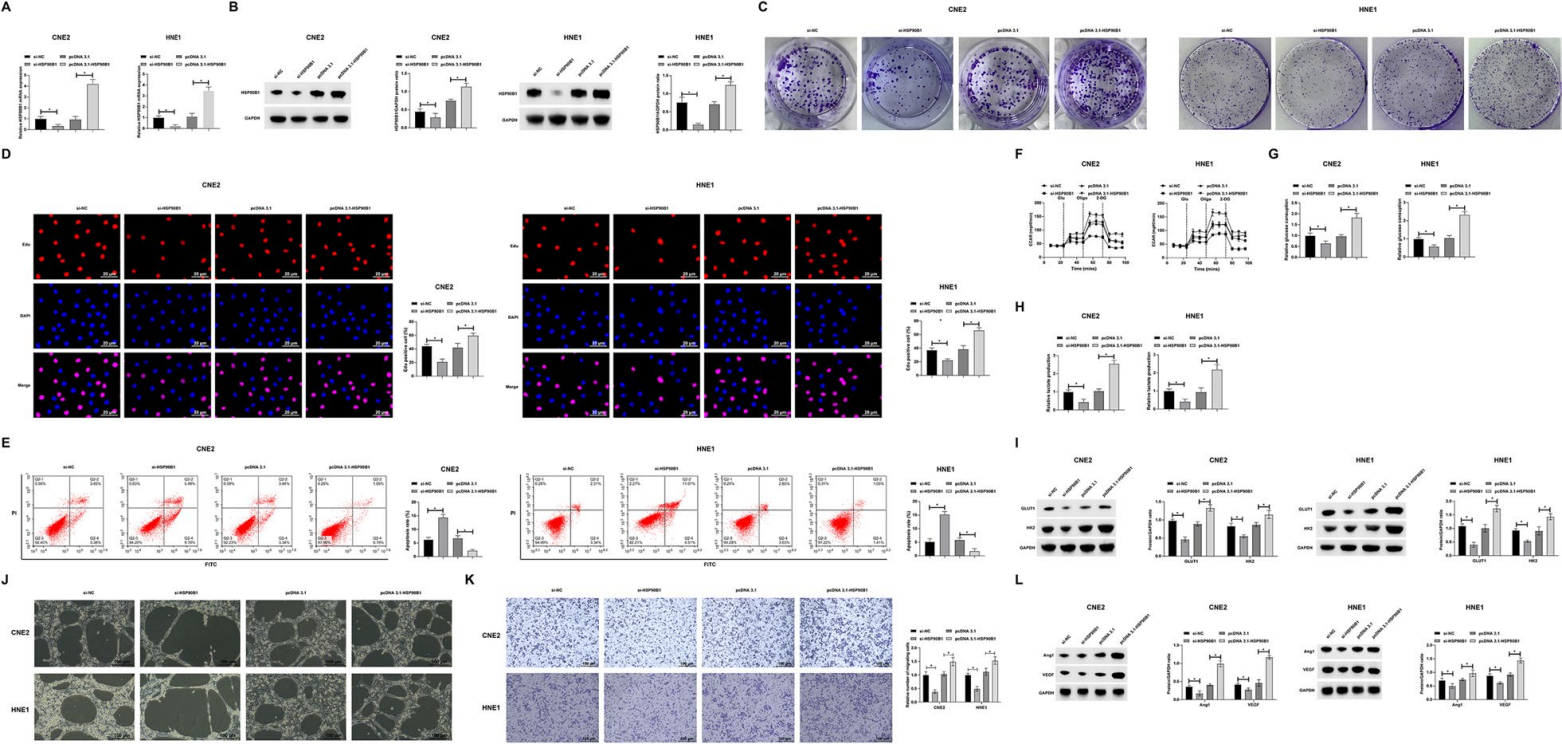
HSP90B1 affects NPC proliferation, glycolysis, and angiogenesis. The siRNA targeting HSP90B1 and pcDNA 3.1 overexpression vector were transfected into HNE1 and CNE2 cells. **A**: RT-qPCR to detect the expression of HSP90B1 in the cells; **B**: Western blot to detect the expression of HSP90B1 in the cells; **C**: Colony formation assay to detect the proliferation of HNE1 and CNE2 cells; **D**: EdU assay to detect proliferation of HNE1 and CNE2 cells; **E**: Flow cytometry to detect apoptosis in HNE1 and CNE2 cells; **F**: Commercial kits to detect ECAR in HNE1 and CNE2 cells; **G**: Commercial kits to detect glucose consumption in HNE1 and CNE2 cells; **H**: Commercial kit to detect lactate production in HNE1 and CNE2 cells; **I**: Western blot to assess GLUT1 and HK2 protein expression in HNE1 and CNE2 cells; **J**: Tube formation assay to detect angiogenic capacity of HUVECs; **K**: HUVEC migration capacity assay; **L**: Western blot to assess the expression of angiogenic proteins Ang-1 and VEGF in HUVECs. Data are expressed as mean ± SD (N = 3). *p* < 0.05 was considered statistically significant

In the realm of NPC, the significant overexpression of HSP90B1 in HNE1 and CNE2 cell lines provided a strategic focus for our investigation. Targeted interventions via siRNA-mediated knockdown and pcDNA 3.1-mediated overexpression of HSP90B1 were implemented. The efficacious transfection was validated, as evidenced in Fig. 2A, B. Assessments of cellular proliferation, utilizing colony formation and EdU incorporation assays, revealed a clear dichotomy: HSP90B1 knockdown significantly impeded, while its overexpression facilitated, the proliferative capacity of NPC cells (Fig. 2C, D). Complementary flow cytometric analysis indicated a direct correlation between HSP90B1 expression levels and apoptotic rates (Fig. 2E). Glycolysis is a major energy source for malignant proliferation and distal metastasis of cancer cells [30]. The metabolic aspect, specifically glycolysis, a pivotal contributor to cancer cell proliferation and metastasis, was also examined. Altering HSP90B1 expression markedly affected ECAR, glucose uptake, lactate production, and the expression of key glycolytic enzymes GLUT1 and HK2 (Fig. 2F–I). This indicated a substantial role of HSP90B1 in metabolic reprogramming within NPC cells. Furthermore, the angiogenic potential of NPC, influenced by HSP90B1 expression, was explored using endothelial cell migration and tube formation assays under various tumor-conditioned media. The results (Fig. 2J, K) illustrated that HSP90B1 knockdown notably hindered, while its overexpression promoted, the angiogenic capabilities of HUVEC cells. This modulation was also reflected in the expression patterns of angiogenesis-related proteins Ang-1 and VEGF (Fig. 2L), underscoring the multifaceted influence of HSP90B1 in NPC pathophysiology.

### Targeted modulation of EGFR expression by HSP90B1 in NPC

**Fig. 3:**
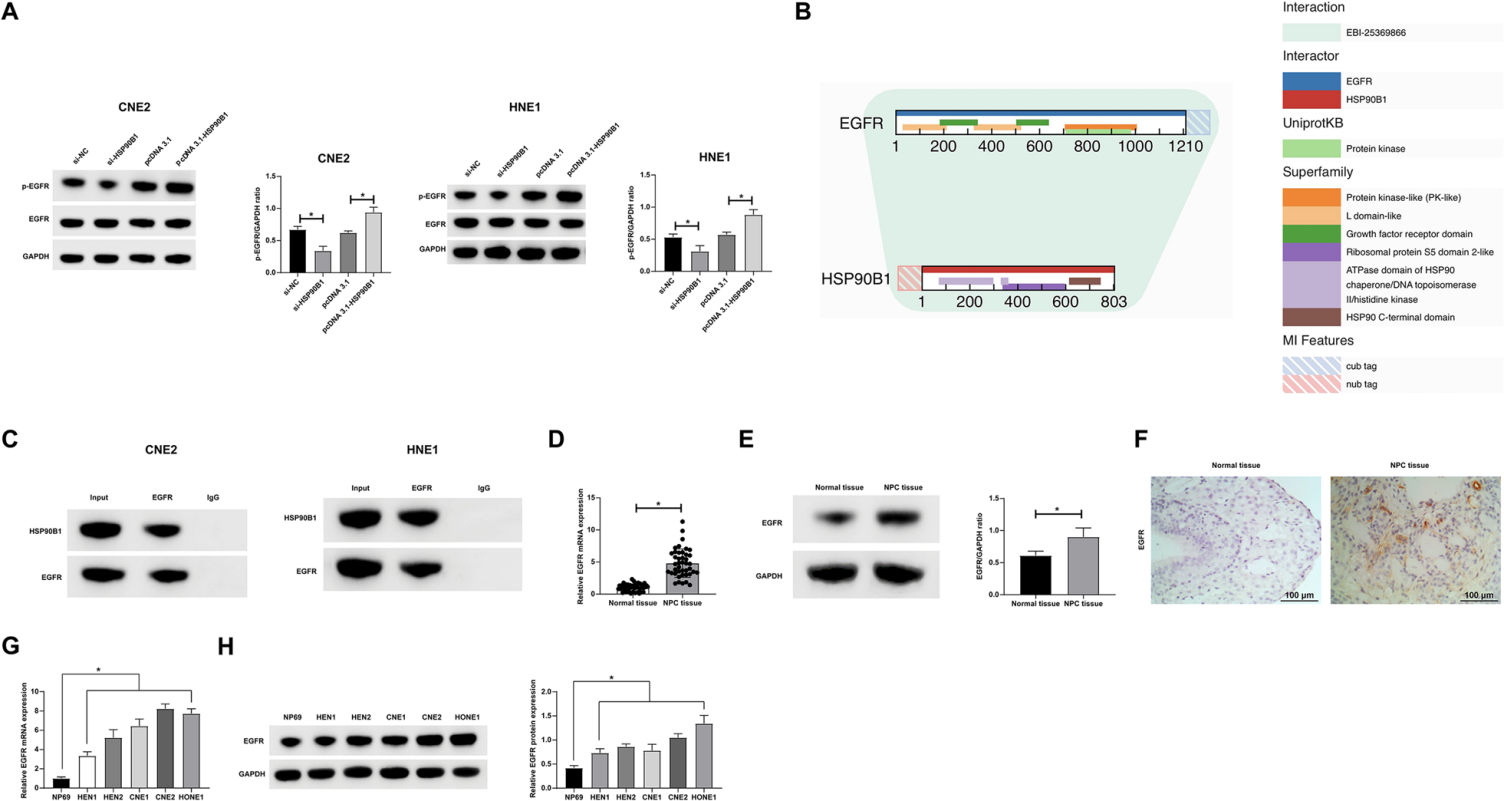
HSP90B1 targets EGFR expression. **A**: Western blot to detect the effect of knockdown or overexpression of HSP90B1 on the expression of phosphorylated EGFR; **B**: Biosignature website (https://www.ebi.ac.uk) to analyze the binding relationship between HSP90B1 and EGFR; **C**: Co-IP assay to detect the interaction between HSP90B1 and EGFR; **D**: RT-qPCR to detect EGFR expression pattern in NPC tissues and normal nasopharyngeal epithelial samples; **E**: Western blot to detect the expression pattern of EGFR in NPC tissues and normal nasopharyngeal epithelial samples; **F**: IHC staining to assess the expression pattern of EGFR in NPC tissues and normal nasopharyngeal epithelial samples; **G**: RT-qPCR to assess the expression pattern of EGFR in NPC cell lines (HNE1, HNE2, CNE1, CNE2, and HONE1) and human immortalized nasopharyngeal epithelial cell line (NP69); **H**: Western blot to assess EGFR expression in NPC cell lines (HNE1, HNE2, CNE1, CNE2, and HONE1) and NP69. Data are presented as mean ± SD (N = 3). *P* < 0.05 was considered statistically significant

Our subsequent investigations delved into the downstream molecular mechanisms of HSP90B1 in NPC. Utilizing bioinformatics resources (http://www.reactome.org), we identified six potential downstream binding proteins of HSP90B1, namely FANCC, HSPA5, APP, EGFR, OS9, and COL1A1. Western blot analyses narrowed down the influence of HSP90B1 specifically to EGFR in NPC cells (Fig. 3A), suggesting a targeted regulatory interaction between HSP90B1 and EGFR (Fig. 3B). This hypothesized interaction was substantiated through Co-IP experiments, confirming the targeted relationship between EGFR and HSP90B1 (Fig. 3C). Additionally, aberrantly high expression of EGFR was observed both in the NPC patient samples and cell lines under study (Fig. 3D–H). These findings collectively indicate a targeted regulatory role of HSP90B1 on EGFR expression in NPC, underscoring a critical aspect of its molecular pathogenesis.

### Deciphering the role of HSP90B1 in EGFR-mediated NPC pathogenesis

**Fig. 4:**
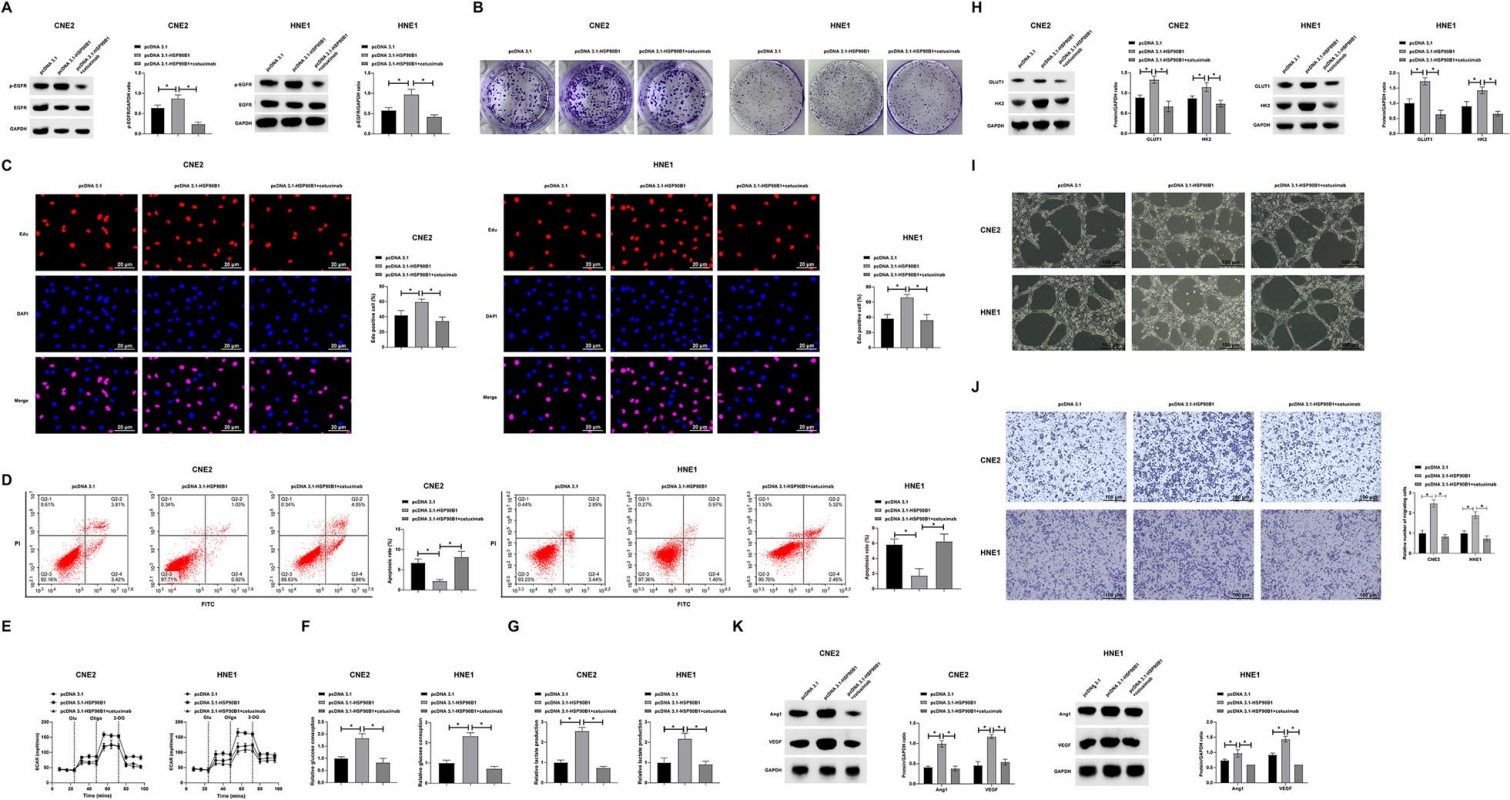
HSP90B1 by regulating EGFR affects NPC malignancy. HNE1 and CNE2 cells transfected with pcDNA 3.1-HSP90B1 were treated with an EGFR inhibitor (cetuximab). **A**: Western blot to detect the phosphorylation level of EGFR; **B**: Colony formation assay to detect proliferation of HNE1 and CNE2 cells; **C**: EdU assay to detect proliferation of HNE1 and CNE2 cells; **D**: Flow cytometry assay to detect apoptosis in HNE1 and CNE2 cells; **E**: Commercial kits to detect ECAR in HNE1 and CNE2 cells; **F**: Commercial kits to detect glucose depletion in HNE1 and CNE2 cells; **G**: Commercial kits to detect lactate production in HNE1 and CNE2 cells; **H**: Western blot assessment of HNE1 and CNE2 cells GLUT1 and HK2 protein expression; **I**: Tube formation assay to detect angiogenic capacity of HUVECs; **J**: HUVEC migration capacity assay; **K**: Western blot to assess the expression of angiogenic proteins Ang-1 and VEGF in HUVECs. Data are presented as mean ± SD (N = 3). *P* < 0.05 was considered statistically significant

In our pursuit to understand the influence of HSP90B1 on NPC, we explored its role in modulating EGFR signaling. Employing cetuximab, an EGFR-specific inhibitor, we aimed to dissect the effects of HSP90B1 overexpression, achieved through pcDNA 3.1 vector transfection, on the EGFR pathway. As shown in Fig. 4A, HSP90B1 overexpression markedly increased EGFR phosphorylation, which was substantially diminished following cetuximab treatment. This modulation of EGFR signaling was further reflected in cellular proliferation dynamics, with colony formation and EdU assays indicating enhanced proliferation in HSP90B1 overexpressing cells, an effect reversed by cetuximab (Fig. 4B, C). Additionally, flow cytometric analysis revealed a decrease in apoptosis in cells overexpressing HSP90B1, a trend inverted by cetuximab treatment (Fig. 4D). The metabolic impact of HSP90B1 overexpression was also evident in enhanced glycolytic activity, as indicated by increased expression of GLUT1 and HK2, effects that were neutralized by cetuximab (Fig. 4E–H). Furthermore, the angiogenic capacity of NPC cells, along with the expression of angiogenesis markers Ang-1 and VEGF, was significantly augmented in the context of HSP90B1 overexpression, with these effects being effectively counteracted in the presence of cetuximab (Figs. 4I–K). These results collectively elucidate a critical mechanism by which HSP90B1 influences NPC pathogenesis through EGFR pathway modulation.

### *HSP90B1 activates the PI3K/Akt/mTOR pathway in NPC cells *via* EGFR regulation*

**Fig. 5:**
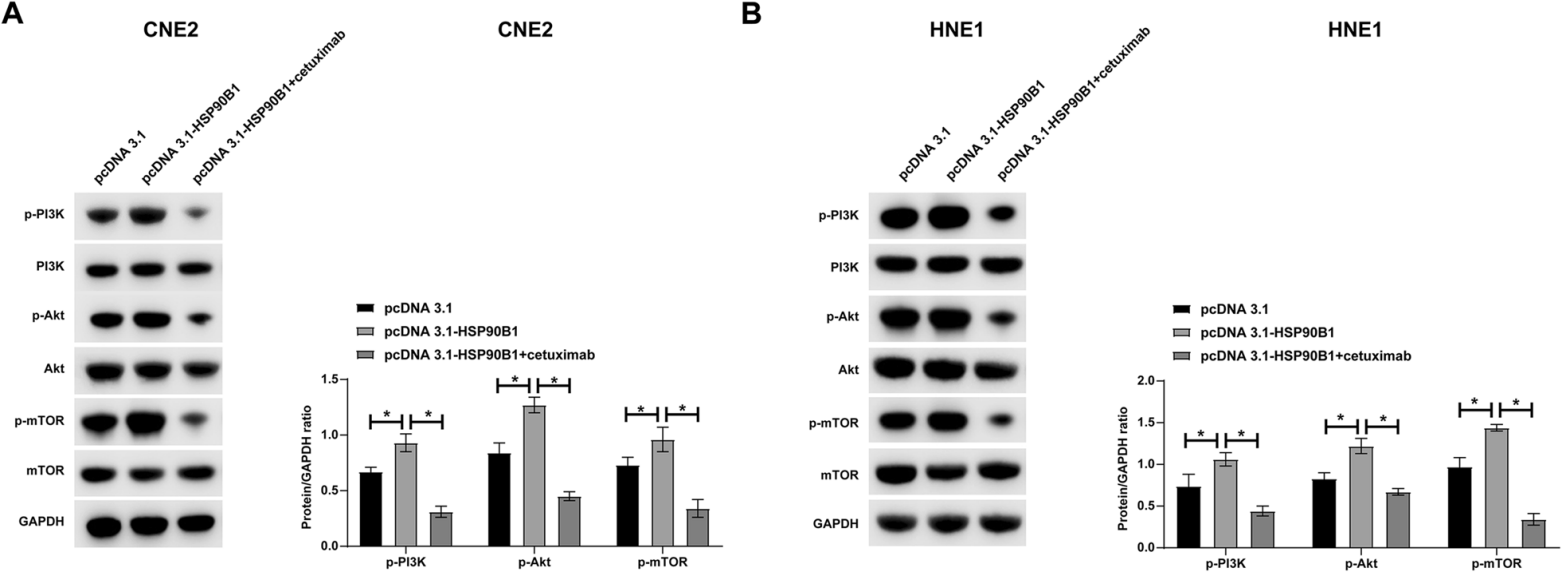
HSP90B1 activates the PI3K/Akt/mTOR pathway in NPC cells by regulating EGFR. HNE1 and CNE2 cells transfected with pcDNA 3.1-HSP90B1 were treated with an EGFR inhibitor (cetuximab). **A**–**B**: Western blot assessed the phosphorylation level of PI3K/Akt/mTOR. Data are presented as mean ± SD (N = 3). *P* < 0.05 was considered statistically significant

Our research probes the mechanistic role of HSP90B1 in regulating the PI3K/Akt/mTOR pathway, a key downstream effector of EGFR signaling implicated in the oncogenesis of various cancers [31, 32]. Specifically, we investigated the potential of HSP90B1 to activate this pathway in NPC cells by modulating EGFR. Western blot analyses provided compelling evidence: Overexpression of HSP90B1 markedly elevated the phosphorylation levels of PI3K/Akt/mTOR components in NPC cells. Crucially, this enhancement was abrogated by cetuximab, an EGFR inhibitor, underscoring the regulatory influence of HSP90B1 on EGFR and consequent activation of the PI3K/Akt/mTOR pathway (Fig. 5A, B).

### FKC inhibits proliferation, glycolysis, and angiogenesis in NPC by targeting HSP90B1

**Fig. 6:**
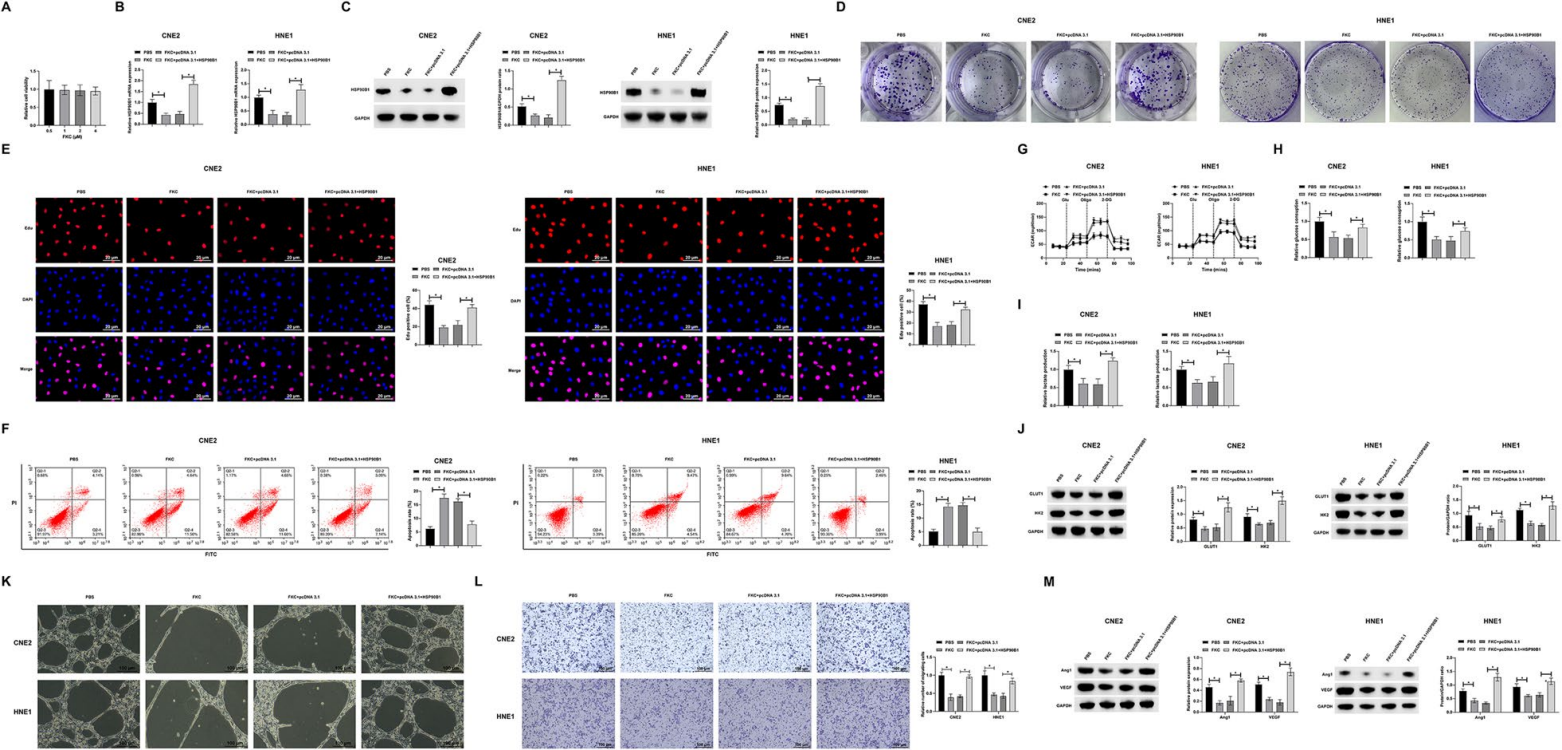
FKC by inhibiting HSP90B1 limits NPC proliferation, glycolysis, and angiogenesis. FKC-treated HNE1 and CNE2 cells were transfected with pcDNA 3.1-HSP90B1. **A**: CCK-8 assay to assess the effect of different concentrations of FKC treatment on NP69 cell viability; **B**: RT-qPCR to detect the expression of HSP90B1; **C**: Western blot to detect the expression of HSP90B1; **D**: Colony formation assay to detect HNE1 and CNE2 cell proliferation; **E**: EdU assay to detect HNE1 and CNE2 cell proliferation; **F**: Flow cytometry to detect HNE1 and CNE2 cell apoptosis; **G**: Commercial kits to detect ECAR in HNE1 and CNE2 cells; **H**: Commercial kits to detect glucose consumption in HNE1 and CNE2 cells; **I**: Commercial kits to detect HNE1 and lactate production in CNE2 cells; **J**: Western blot assessment of GLUT1 and HK2 protein expression in HNE1 and CNE2 cells; **K**: Tube formation assay to detect the angiogenic capacity of HUVECs; **L**: Migration capacity assay of HUVECs; **M**: Western blot assessment of angiogenic proteins Ang-1 and VEGF in HUVEC. Data are presented as mean ± SD (N = 3). *P* < 0.05 was considered statistically significant

Our previous research has identified FKC as a potent HSP90B1 inhibitor, manifesting significant anti-proliferative properties in NPC cells. Herein, we explored whether FKC exerts its effects by modulating HSP90B1, thereby affecting NPC cell proliferation, glycolysis, and angiogenesis. Initially, we evaluated FKC's cytotoxicity on normal nasopharyngeal epithelial cells (NP69). Figure 6A demonstrates that varying concentrations of FKC treatment for 24 h did not adversely affect NP69 cell viability. In HNE1 and CNE2 cells treated with FKC, pcDNA 3.1-HSP90B1 transfection was performed. Results, as shown in Fig. 6B, C, indicate that FKC treatment reduced HSP90B1 expression, which was effectively countered by the introduction of pcDNA 3.1-HSP90B1. Through colony formation and EdU assays, we observed that FKC treatment notably inhibited cell proliferation, an effect mitigated by HSP90B1 overexpression (Fig. 6D, E). Flow cytometric analysis revealed an increase in apoptotic rates following FKC treatment, which was reversed upon overexpression of HSP90B1 (Fig. 6F). Furthermore, FKC treatment led to a reduction in glycolytic activity and downregulation of the glycolysis-related proteins GLUT1 and HK2, effects that were reversed with HSP90B1 overexpression (Fig. 6G–J). In addition, FKC impaired angiogenesis in NPC cells and reduced the expression of angiogenic proteins Ang-1 and VEGF, which was subsequently restored with HSP90B1 overexpression (Fig. 6K–M). Collectively, these data indicate that FKC modulates NPC cell behavior by targeting HSP90B1, leading to inhibition of proliferation, glycolysis, and angiogenesis.

### *FKC modulates the PI3K/Akt/mTOR pathway in NPC cells *via* HSP90B1 regulation*

**Fig. 7:**
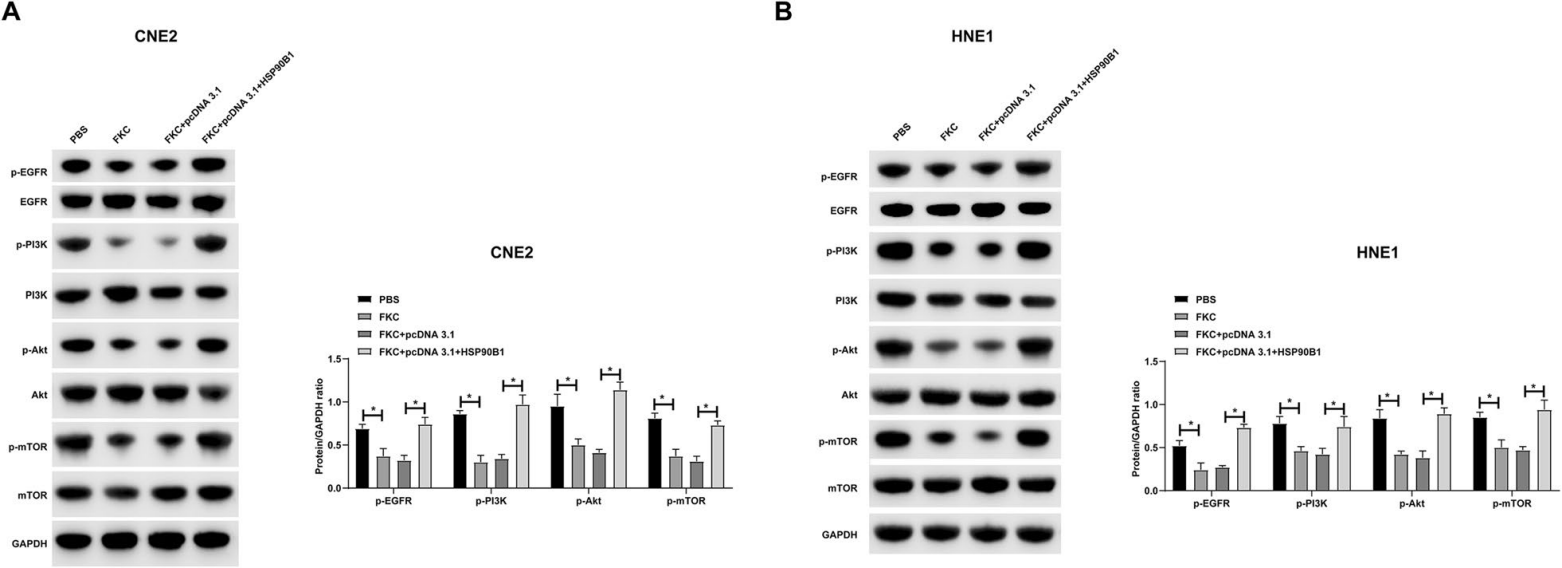
FKC by inhibiting HSP90B1 affects the PI3K/Akt/mTOR pathway in NPC cells. pcDNA 3.1-HSP90B1 was transfected into FKC-treated HNE1 and CNE2 cells. **A**–**B**: Western blot detected phosphorylation levels of PI3K/Akt/mTOR. Data are presented as mean ± SD (N = 3). *P* < 0.05 was considered statistically significant

We hypothesized that FKC may modulate the PI3K/Akt/mTOR pathway in NPC cells by regulating the expression of HSP90B1. To test this hypothesis, we conducted Western blot analyses. The results revealed that treatment with FKC led to a decrease in the phosphorylation levels of EGFR/PI3K/Akt/mTOR in NPC cells. Importantly, this effect was reversed upon overexpression of HSP90B1 (Fig. 7A, B). These findings suggest that FKC impacts the activation of EGFR, thereby modulating the PI3K/Akt/mTOR pathway, through its regulatory effect on HSP90B1.

### FKC modulates tumor growth and metastasis in NPC through the HSP90B1/EGFR axis

**Fig. 8:**
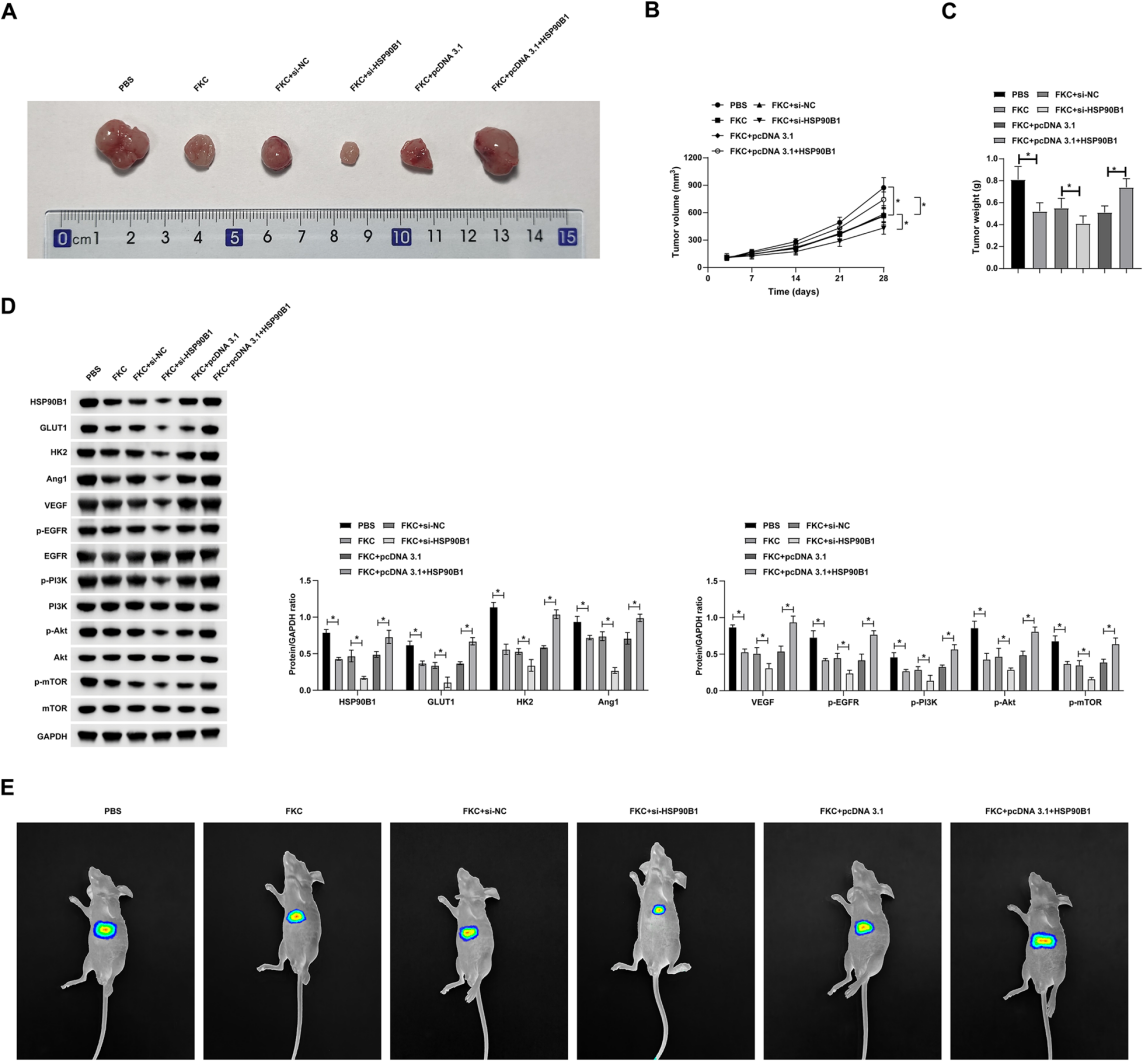
FKC by modulating the HSP90B1/EGFR axis affects NPC tumor growth and metastasis. **A**: Tumor images; **B**: Tumor volume; **C**: Tumor weight; **D**: Western blot assessment of relevant protein expression in tumors; **E**: Noninvasive bioluminescence imaging of luciferase-expressing intrahepatic HNE1 cell xenografts showing FKC inhibition of in situ liver metastatic tumor formation. Data are presented as mean ± SD (n = 5). *P* < 0.05 was considered statistically significant

In the next phase of our study, we employed xenograft models in nude mice to assess the effect of FKC on the in vivo progression and metastatic potential of NPC. The results, depicted in Fig. 8A–C, showed that FKC treatment led to a marked reduction in both tumor volume and weight. Notably, this reduction was further accentuated by the knockdown of HSP90B1, while overexpression of HSP90B1 mitigated the inhibitory effects of FKC. Furthermore, FKC treatment resulted in the downregulation of HSP90B1, GLUT1, HK2, Ang-1, and VEGF, and concurrently inhibited the phosphorylation of EGFR/PI3K/Akt/mTOR. This suppression was intensified by HSP90B1 knockdown but reversed with its overexpression (Fig. 8D). Additionally, FKC effectively inhibited liver metastasis of NPC tumors (Fig. 8E, F). These findings collectively demonstrate that FKC exerts its anti-tumorigenic and anti-metastatic effects in NPC through modulation of the HSP90B1/EGFR pathway.

## Discussion

HSP90 family has been widely reported to affect many oncogene pathways and regulate tumor cell growth and survival [18, 33]. Therefore, HSP90 family has become the forefront and hot spot of anti-tumor drug research. FKC, a natural anticancer in Chinese medicine that can target multiple molecular pathways, has shown considerable efficacy in colon cancer [17, 28]. Previous studies have demonstrated that FKC is a natural inhibitor of HSP90B1, which can effectively reduce the expression of HSP90B1 in cancer cells. However, it is not clear whether FKC can influence the malignant behavior of NPC by regulating HSP90B1 and its downstream molecular pathways. This study found that FKC acts as an inhibitor of HSP90B1 to block the EGFR pathway, thereby inhibiting NPC proliferation, invasion, migration, and glycolysis.

HSP90B1 is a subgroup of HSP90, also named Grp94 (94 kDa glucose-regulatory protein), which is located in the ER lumen and secreted in the cell membrane [20]. Under low glucose and hypoxia, more misfolded and denatured proteins accumulate in the endoplasmic reticulum, a condition known as endoplasmic reticulum stress (ERS). In the ERS environment, HSP90B1 is activated, folds and assembles proteins, and promotes cell survival [20, 34]. Recent studies have observed that high expression of HSP90B1 has prognostic significance in some malignant tumors [20–22]. A recent study also shows that HSP90B1 plays a greater role in the carcinogenesis of breast cancer [22]. In osteosarcoma, silencing HSP90B1 achieves tumor-suppressing effects on tumor growth, possibly through modulating the PI3K/AKT/mTOR pathway [35]. This study also found that HSP90B1 was highly expressed in NPC cells and cancer tissues. Knockdown of HSP90B1 inhibited the proliferation, invasion, migration, glycolysis, and angiogenesis of NPC cells, while overexpression of HSP90B1 had the opposite effect. In addition, through correlation analysis of clinicopathological features, it was found that high level of HSP90B1 expression was positively correlated with lymph node metastasis and distant metastasis in patients. The promotion of high levels of HSP90B1 on NPC cell proliferation, glycolysis, and angiogenesis may be the main cause of cancer cell metastasis. Notably, high levels of HSP90B1 are associated with poor prognosis in a variety of cancers [36, 37]. In this study, it was also found that NPC patients with high expression of HSP90B1 had worse survival prognosis. Therefore, targeted regulation of HSP90B1 may pay attention to improving the survival rate of NPC patients.

Since the molecular mechanism of HSP90B1 regulating NPC is still unclear, this study then explored the downstream molecular targets of HSP90B1. This study analyzed potential downstream targets of HSP90B1 from the bioinformatics website http://www.reactome.org and confirmed that EGFR is the downstream target gene of HSP90B1. EGFR has been shown to be overexpressed in cancer, and EGFR antagonists have been used in cancer therapy [38, 39], which is consistent with the results of this study. In addition, overexpression of HSP90B1 was found to promote EGFR phosphorylation. However, the promotion effect of overexpression of HSP90B1 on malignant behavior of NPC cells was reversed by EGFR inhibitors. This indicates that HSP90B1 is targeted to activate EGFR to regulate NPC biological behavior.

EGFR is a cell surface tyrosine kinase receptor that can recruit and phosphorylate cytoplasmic signaling molecules in an activated condition, and thereby initiate downstream signaling cascades, including PI3K/AKT, to promote tumor cell proliferation [40, 41]. Notably, EGFR overexpression downregulates intracellular ROS levels via the PI3K/AKT pathway, thereby accelerating the metastasis potential of NPC cells [41]. Interestingly, the present study discovered that HSP90B1 promoted PI3K/Akt/mTOR phosphorylation in NPC cells, but this effect was rescued by an EGFR inhibitor (cetuximab). This indicates that HSP90B1 can regulate EGFR to activate the PI3K/Akt/mTOR pathway of NPC cells, which will have an important impact on the biological behavior of NPC cells.

FKC belongs to the flavonoid family, whose anticancer properties have been extensively studied [13]. Several studies have reported the inhibitory effect of flavonoids on the malignant behavior of NPC, such as Baicalein and isoliquiritigenin [42, 43]. In the present study, we found that treatment of NPC cells with another flavonoid, FKC, effectively prevented NPC cell proliferation, triggered apoptosis, and reduced glycolysis and angiogenesis, which was reversed by overexpression of HSP90B1. Notably, FKC had only minor toxic effects on normal cells [16]. In addition, flavonoid Trifolirhizin can block the PI3K/Akt pathway [44]. Similar results were obtained in the present study. Phosphorylation levels of EGFR/PI3K/Akt/mTOR were reduced in NPC cells after FKC treatment, but this effect was reversed by overexpression of HPS90B1. To further validate the results of our in vitro study experiments, nude mouse xenograft experiments were performed to assess the effects of FKC on NPC tumor growth and metastasis in vivo. It was found that FKC treatment suppressed tumor volume and weight and inhibited glycolysis and angiogenesis-related protein expression and phosphorylation of the EGFR/PI3K/Akt/mTOR pathway, whereas knockdown of HSP90B1 strengthened the effect of FKC. In addition, FKC inhibited liver metastasis of NPC tumors. These findings confirm that FKC affects the growth and metastasis of NPC by regulating HSP90B1 to influence EGFR activation and modulating the PI3K/Akt/mTOR pathway.

When exploring the possible mechanisms of resistance to FKC in NPC treatment, we must take into account the complex adaptations and survival strategies of cancer cells. First, tumor cells may reduce drug sensitivity by altering the structure or expression of HSP90B1, the primary target of FKC. This protein alteration may affect the ability of FKC to regulate EGFR phosphorylation, which in turn affects the PI3K/Akt/mTOR pathway, leading to a diminished anticancer effect of FKC. Second, changes in intracellular drug metabolism are also a key factor contributing to drug resistance [45]. Tumor cells may accelerate the metabolism of FKC and reduce its effective intracellular concentration by enhancing the expression or activity of drug metabolizing enzymes. In addition, overexpression of drug pump proteins, such as the upregulation of P-glycoprotein, may increase cellular exclusion of FKC, further decreasing drug effectiveness [46]. In addition, cancer cells may escape FKC-induced cell death by activating other survival signaling pathways or inhibiting apoptotic pathways. For example, by activating alternative growth factor receptors or regulating the expression of apoptosis-related proteins [47, 48] cells may develop resistance to FKC. Finally, genetic heterogeneity and clonal evolution of tumors are also important factors in the development of drug resistance [49, 50]. There may be subpopulations of tumor cell populations that are naturally resistant to FKC, and these cells may be screened out during treatment, leading to an overall increase in resistance. Given these potential mechanisms of resistance, future research should focus on developing strategies to overcome or mitigate these challenges, such as through the combination of drugs with different mechanisms of action, implementation of individualized treatment regimens, and the development of new drugs or therapeutic approaches targeting the aforementioned mechanisms of resistance. Through such a multifaceted approach, we can more effectively address the resistance problems that FKC may encounter in NPC treatment.

However, only cell culture investigations and experimental animal studies are not enough to generalize the study findings to patients. Furthermore, the possibility that FKC modulates other transcription factors and signaling pathways cannot be ruled out, which needs to be explored in future studies.

## Conclusion

In summary, our study demonstrates that HSP90B1 is a novel oncogenic factor in NPC. It activates PI3K/Akt/mTOR signaling pathway by promoting EGFR phosphorylation, thus promoting malignant behaviors of NPC. As a new inhibitor of HSP90B1, FKC has a positive effect on preventing NPC from malignant behaviors. The findings provide strong data support for the development of novel therapeutic drugs for NPC and the exploration of molecular targets.

### Supplementary Information

**Additional file1.** Original Image for Western Blots.

## Data Availability

No datasets were generated or analysed during the current study.
